# Ratiometric Polymer Probe for Detection of Peroxynitrite and the Application for Live-Cell Imaging

**DOI:** 10.3390/molecules24193465

**Published:** 2019-09-24

**Authors:** Hio Kuan Lao, Jingyun Tan, Chunfei Wang, Xuanjun Zhang

**Affiliations:** Cancer Centre and Centre of Reproduction, Development and Aging, Faculty of Health Sciences, University of Macau, Macau SAR 999078, China; cb52929@connect.um.edu.mo (H.K.L.); yb67596@connect.um.edu.mo (C.W.)

**Keywords:** ratiometric, fluorescent probe, polymer probe, peroxynitrite, cell imaging

## Abstract

Peroxynitrite (ONOO^−^) is one of the sources of oxidation stress involved in many biological signaling pathways. The role of ONOO^−^ being a double-edged sword in biological systems drives the development of effective detection tools. In this work, a boronate-based polymeric fluorescent probe PB-PVA was synthesized and the probe performance was evaluated. The probe exhibits ratiometric sensing of ONOO^−^ in a range of 0–6 µM. There is good linear relationship between the probe fluorescence intensity ratio and ONOO^−^ concentration. The probe also displays moderate selectivity towards ONOO^−^ over other ROS. Moreover, it is water-soluble and possesses good biocompatibility which aids the imaging of ONOO^−^ in living cells. These properties could make the probe a promising tool in in vitro study related to ONOO^−^.

## 1. Introduction

Both reactive oxygen species (ROS) and reactive nitrogen species (RNS) play a double role in physiological pathways. ROS/RNS functions as crucial signaling molecules involved in various signaling pathways, such as PI3K-Akt and MAPK signaling pathways [1,2,3]. They can transduce various cellular signals and induce stress response [4,5]; however, they are also the source of oxidation stress. They oxidize and damage many biomolecules, such as DNA, lipids, and proteins, leading to cellular dysfunction. One member in the RNS family, peroxynitrite (ONOO^−^), which is produced from a diffusion-controlled reaction between nitric oxide (NO·) and superoxide (O_2_^−^), is a potent oxidizing agent that damages various biomolecules. There is growing evidence on its role in the physiological and pathophysiological pathway in living systems [3,5]. Dysregulated ONOO^−^ levels disrupt cell signaling pathways and contribute to Type 1 diabetes, diabetic cardiovascular disease, and neurodegenerative disorders [5]. Therefore, a fast-responding and reliable ONOO^−^ probe is highly desired for understanding the role of ONOO^−^ in biological system and ONOO^−^-related human disease detection.

Fluorescent probes have been developed and used extensively for biomolecule imaging in recent years. There are many reports on non-ratiometric fluorescent probes for ONOO^−^ detection [6,7]. However, the non-ratiometric measurement can be easily affected by different factors, such as probe concentration, light scattering through the matrix, microenvironment around the probe, and uneven probe distribution [8,9,10]. Ratiometric measurement relies on the intensity changes of two or more emission bands. This method can increase the signal-to-noise ratio and provide a more accurate measurement.

In the design of the probe, boronates are considered since boronates are recognized as one of the most effective peroxynitrite-detecting organic molecules [11]. A boronate signaling dye (BT) in a Förster resonance energy transfer (FRET)-based probe that we previously reported [12] and a newly synthesized dye (EP) are involved in our probe. In addition, polyvinyl alcohol (PVA) is also incorporated. PVA is generally considered safe and biocompatible as it is inert and stable [13,14], together with its high degree of functionality, it is considered as a perfect candidate for the linking molecule of molecular probes. Grafting organic small molecules onto PVA can increase the solubility of the small molecules in water, facilitating the probe delivery into living cells and the application of probe within the water-based biological system.

**Scheme 1:**
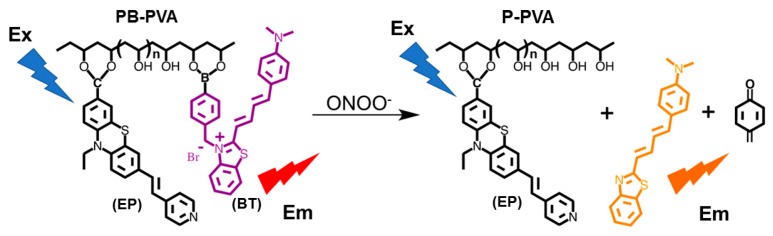
Design and ONOO^−^ sensing mechanism of the probe PB-PVA.

Herein, we report on a boronate-based polymeric fluorescent probe PB-PVA for the ratiometric measurement of ONOO^−^ and the imaging of exogenous ONOO^−^ in vitro. BT and EP were grafted onto PVA to produce PB-PVA. The structure and sensing mechanism are depicted in Scheme 1. This probe exhibits ratiometric sensing of ONOO^−^ with moderate selectivity and fast response. Furthermore, the probe is cell-permeable and displays low cytotoxicity, which facilitates the probe application in ONOO^−^-related in vitro studies.

## 2. Results and Discussion

### 2.1. Synthesis of the Probe PB-PVA

**Scheme 2:**
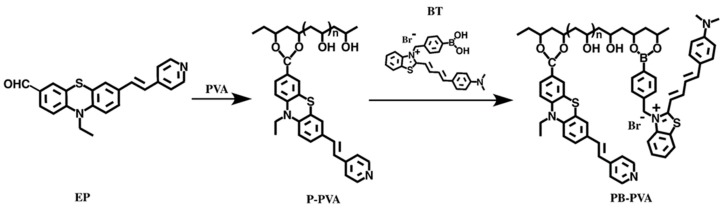
Synthesis route of PB-PVA.

The probe was obtained by grafting EP and BT onto PVA in two steps (Scheme 2), first to produce PVA grafted by EP (P-PVA), followed by grafting BT onto P-PVA.

EP was grafted on PVA to produce P-PVA through acetalization with the modified published method [15]. EP (50.0 mg, 0.10 mmol) and PVA (36.8 mg, 13,000–23,000 Da) and p-toluenesulfonic acid (p-TSA) (15.9 mg, 0.09 mmol) in DMSO was heated to 80 °C for 48 h. Dialysis was then performed in water to remove the free EP and DMSO. The grafting ratio was determined by ^1^H NMR. The ratio of EP grafted to corresponding monomer of PVA is 1:10. (Figure S6).

**Figure 1:**
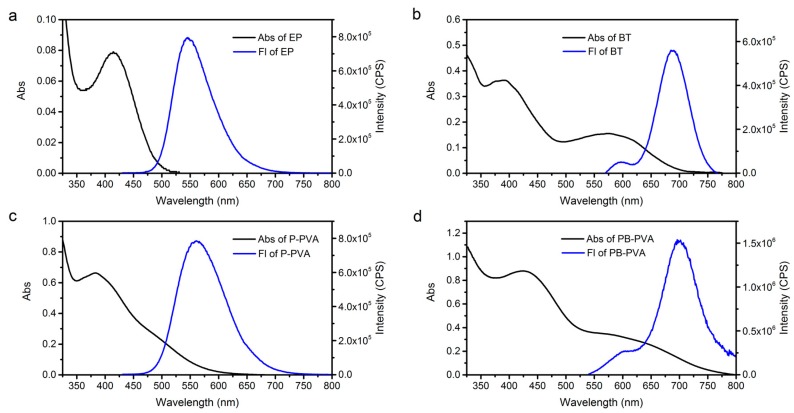
The absorption and fluorescence spectra of (**a**) EP, (**b**) BT, (**c**) P-PVA, and (**d**) PB-PVA.

In order to obtain PB-PVA, BT and P-PVA (with EP grafted and BT in a molar ratio of 1:4) was stirred in DMSO at dark environment in room temperature for five days. Dialysis was then performed in water to remove the free BT and DMSO. The ratio of EP and BT grafted onto PVA was determined by absorption spectrum and the ratio is estimated to be 1.6:1 (Figure 1d). The final grafting ratio of PVA monomer to EP grafted to BT grafted is estimated to be 16:1.6:1. In the study, the probe concentration refers to the concentration of BT grafted in the probe solution. Unless otherwise stated, the PB-PVA sample solution (10 µM) in 20% (*v*/*v*) of DMSO in water was used in the optical tests.

### 2.2. Optical Properties of the Intermediates and PB-PVA

The basic optical properties of the two newly synthesized small molecules EP and BT were investigated. EP has peak absorption at 414 nm with extinction coefficient 15,600 M^−1^ cm^−1^ and strong emission at 550 nm with excitation at 420 nm (Figure 1a). BT has peak absorption at 563 nm with extinction coefficient 11,300 M^−1^ cm^−1^ and strong emission at 700 nm with excitation at 560 nm (Figure 1b). It was reported that BT has good sensing performance towards ONOO^−^, the emission peak at 700 nm is replaced by new band at 540 nm when BT is reacted completely with ONOO^−^ [12]. PB-PVA has two emission peaks with excitation at 500 nm, one at 600 nm and one at 700 nm (Figure 1d).

### 2.3. Ratiometric Fluorescence Response of PB-PVA to ONOO^−^

**Figure 2:**
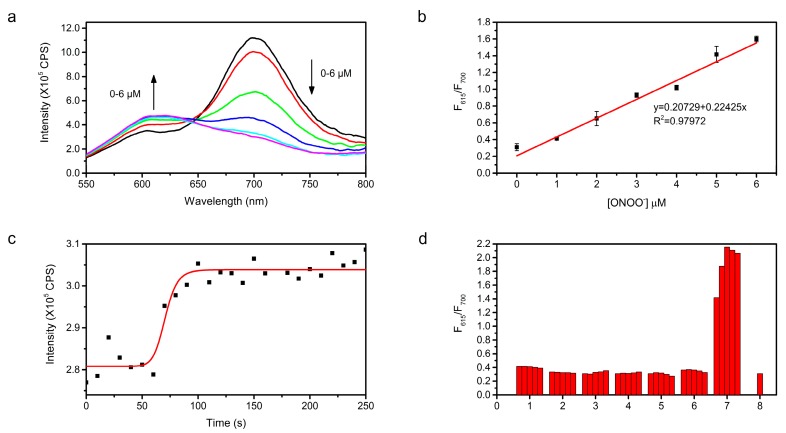
(**a**) Fluorescence spectrum of PB-PVA in response to ONOO^–^ at different concentrations in water with 20% *v*/*v* of DMSO. λ_ex_ = 500 nm. (**b**) F_615_/F_700_ vs. ONOO^–^ concentration from 0 to 6 μM. The values are expressed as mean ± standard deviation of three independent experiments. (**c**) The time course of fluorescence intensity of PB-PVA (10 μM) at 600 nm after adding ONOO^−^ (100 μM). (**d**) Selectivity of PB-PVA (10 μM) to various analytes (1. ClO^−^, 2. H_2_O_2_, 3. NO·, 4. NO_2_^−^, 5. NO_3_^−^, 6. Cys, 7. ONOO^−^ at 5, 10, 20, 50, 100 μM; 8. Blank).

The excitation of PB-PVA at 500 nm resulted in two emission peaks, one from the EP grafted at 600 nm and one from the BT grafted at 700 nm. With the addition of increasing concentration of ONOO^−^, the peak at 615 nm rose while the one at 700 nm decreased in a regular manner (Figure 2a). Since the boronate group on BT is easily degraded by ONOO^−^, the decrease in the fluorescence intensity at 700 nm represents the declining amount of the BT grafted on the probe due to ONOO^−^ exposure. The product from the BT grafted degradation gave a yellowish orange emission, responsible for the increasing peak at 615 nm (Figure 2a). The fluorescence intensity ratio (F_615_/F_700_) from exposing the probe to 0–80 μM of ONOO^−^ was then calculated (Figure S1) and it shows good linear relationship with ONOO^−^ concentration ranging from 0 to 6 μM (Figure 2b). The F_615_/F_700_ exhibited five-fold enhancement from 0.31 before the exposure to 1.60 upon the addition of 6 μM of ONOO^−^. The linear regression equation is y = 0.20729 + 0.22425x with correlation coefficient R^2^ = 0.97972 (Figure 2b). The model suggests this probe can be applied for ratiometric sensing of ONOO^−^ within the concentration of 0–6 μM. The detection limit is calculated as 0.30 μM by the equation 3σ/k (n = 15). To evaluate the response time, the emission at 600 nm was tracked immediately after the addition of ONOO^−^ and the fluorescence intensity became stable after 50 s (Figure 2c). The probe could give fast response towards ONOO^−^ within 1 min. The probe was subsequently exposed to different ROS to investigate the selectivity toward ONOO^−^. Interfering species which the probe may encounter in the biological system, such as ClO^−^, H_2_O_2_, NO·, NO_2_^−^, NO_3_^−^, and cysteine (Cys) in a wide range of concentrations (0–100 μM) were exposed to the probe. The F_615_/F_700_ from ONOO^−^ exposure was the most significant compared with other ROS exposure, implying the probe exhibits good selectivity towards ONOO^−^ and is not easily affected by the mentioned interfering species (Figure 2d). As we know, peroxynitrite is active (within the millisecond range) and the in vivo concentration is low (within micromole range) [16,17] and the probe PB-PVA shows potential in biological applications.

### 2.4. Confocal Laser Scanning Microscope (CLSM) Imaging of ONOO^–^ in HeLa Cells

**Figure 3:**
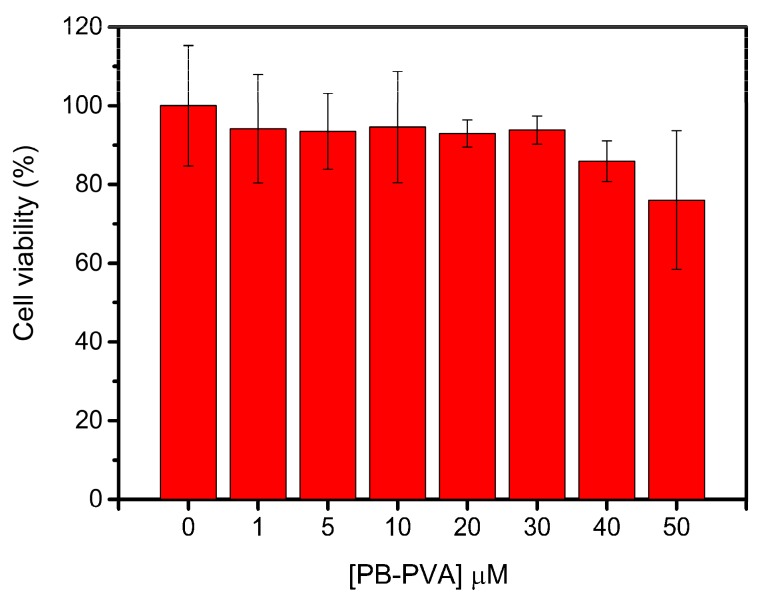
MTT assay for estimating cell viability (%) of HeLa cells treated with various concentration of PB-PVA (5–50 μM) for 20 h. The values are expressed as the mean ± standard deviation of three independent experiments.

Cytotoxicity assay was performed before cell imaging experiments. The probe shows low cytotoxic effect with cell viability over 90% at 30 μM in MTT assay (Figure 3).

**Figure 4:**
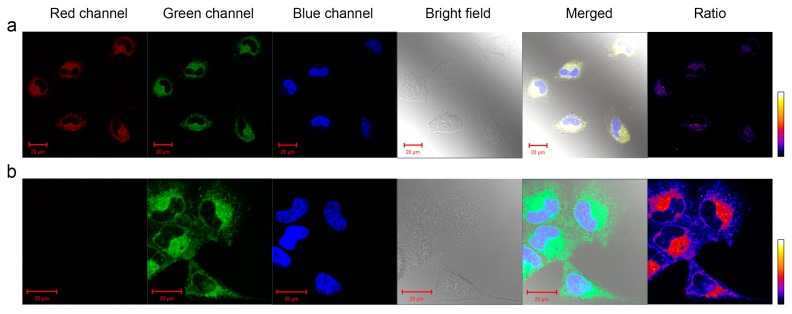
CLSM images of HeLa cells. (**a**) HeLa cells were treated with PB-PVA (5 μM) for 2 h; (**b**) Cells pretreated with PB-PVA (5 μM) were further treated with 1 mM of SIN-1 for 1 h. Red channel: λ_ex_ = 633 nm, λ_em_ = 640–740 nm; green channel: λ_ex_ = 488 nm, λ_em_ = 530–615 nm; blue channel: λ_ex_ = 405 nm, λ_em_ = 410–495 nm (the ratio was calculated by the intensity of green channel over the red channel).

The feasibility of applying the probe PB-PVA for in vitro study involving complex biological environment was then evaluated. HeLa cells were incubated with PB-PVA (5 μM) in order to investigate the cellular permeability of the probe. The HeLa cells could take up the polymeric probe and fluorescence could be observed within the cells from the green channel (530–615 nm) and red channel (640–740 nm) (Figure 4a). The nucleus was stained by Hoechst 33342 (blue channel). The result shows that the probe locates in cytoplasm. Hela cells pre-incubated with the probe were then exposed to exogenous ONOO^−^ produced by ONOO^−^ generator 3-morpholinosydnonimine (SIN-1). The fluorescence signal from red channel, representing the presence of BT, was significantly weakened after SIN-1 treatment (Figure 4b). The signal from the green channel was also enhanced from the probe degradation product. To quantify the cell imaging results, the fluorescence signal ratio between green channel and red channel was calculated via the software ImageJ. The ratio for the mean intensity of the probe over the probe reacted with ONOO^−^ was 1.3 and 6.6, respectively, showing about five-fold enhancement. These results from CLSM imaging show that this probe is cell permeable and can response to ONOO^−^ in vitro for exogenous ONOO^−^ imaging.

## 3. Experimental Section

### 3.1. General

All commercially available reagents were purchased from Sigma-Aldrich (Sigma-Aldrich Corporation, St. Louis, MO, USA), and were used without purification. All solvents for reactions and spectral measurement were purified by conventional methods before use. Hoechst 33342 (cas: 23491-52-3) for cell nucleus staining was purchased from Thermo (ThermoFisher Scientific, Waltham, MA, USA). The peroxynitrite donor SIN-1 was purchased from Sigma-Aldrich (Sigma-Aldrich Corporation, St. Louis, MO, USA). RPMI 1640 medium for cell incubation was purchased from Gibco (Gibco, Grand Island, NY, USA). The NMR spectra were recorded at 25 °C on a Bruker Avance 400 spectrometer (Bruker Biospin, Rheinstetten, Germany), and the chemical shifts are reported as parts per million from TMS (δ). Coupling constant J is given in Hertz. Mass spectra were determined with the ESI mass spectra, which were recorded on a Waters Xevo G2-XS QT matrix-assisted time of flight mass spectrometer (Waters, Milford, MA, USA). UV–VIS absorption spectra were recorded on a SHIMADZU UV-1800 spectrophotometer (Shimadzu, Japan). Fluorescence spectra were performed on Horiba Jobin–Yvon Fluorolog-3 modular spectrofluorometer (HORIBA Scientific, Edison, NJ, USA). The cell images were detected using LSM 700 Zeiss confocal microscope (Carl Zeiss, Oberkochen, Germany), and processed with software ImageJ (NIH, Bethesda, MD, USA).

### 3.2. Synthesis and Characterization of EP and BT

**Scheme 3:**
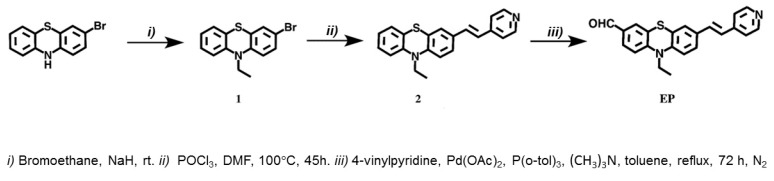
Synthesis route of EP.

**Scheme 4:**
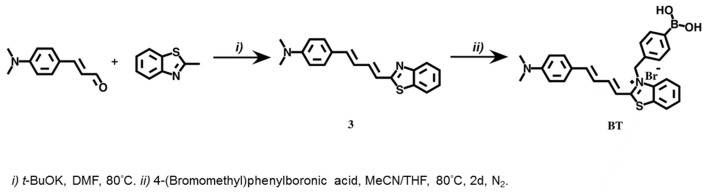
Synthesis route of BT.

The synthesis route of EP and BT is shown in Scheme 3 and Scheme 4. The intermediates and the probe were characterized, the detailed characterization information is given in the Supplementary Information.

#### 3.2.1. Synthesis of 3-bromo-10-ethyl-10*H*-phenothiazine (Compound **1**)

Compound **1** was synthesized by the modification of the published method [18]. Sodium hydride (2.2 g, 53.9 mmol) was stirred with 3-bromo-10*H*-phenothiazine (10.0 g, 35.9 mmol, dissolved in dry DMF) for 30 min. Bromoethane (4.7 g, 43.1 mmol) was then added to the mixture and stirred for 10 h at room temperature in dark environment. After the reaction completed, water was added to react the remaining sodium hydride. White solid was precipitated and collected by vacuum filtration. The solid was first washed with small amount of EA and then washed with n-hexane. ^1^H NMR (400 MHz, acetone-*d*_6_) δ 7.34 (dd, *J* = 8.7, 2.3 Hz, 1H), 7.28 (d, *J* = 2.3 Hz, 1H), 7.25–7.19 (m, 1H), 7.15 (dd, *J* = 7.6, 1.5 Hz, 1H), 7.04 (dd, *J* = 8.3, 1.2 Hz, 1H), 6.99 (d, *J* = 1.2 Hz, 0H), 6.97 (s, 1H), 6.95 (s, 1H), 3.99 (q, *J* = 6.9 Hz, 2H), 1.38 (t, *J* = 6.9 Hz, 3H).

#### 3.2.2. Synthesis of 7-Bromo-10-ethyl-10H-phenothiazine-3-carbaldehyde (Compound **2**)

Compound **2** was synthesized by the modification of the published method [18]. Phosphorus oxychloride (16.3 mL, 106.0 mmol) was added dropwise in cold dry DMF (7.8 g, 106.1 mmol) on ice-salt bath over a period of 2.5 h. Compound **1** (6.5 g, 21.2 mmol) was then added to the solution and heated to 100 °C for 45 h. The resulting mixture was cooled to room temperature and poured into ice water. The solution was neutralized to pH 7–8 and extracted with DCM/water. Silica gel column chromatography was run to purify the product by using n-hexane and DCM (*v*/*v* = 2:1). ^1^H NMR (400 MHz, acetone-d6) δ 9.80 (s, 1H), 7.67 (dd, *J* = 8.5, 1.9 Hz, 1H), 7.50 (d, *J* = 1.9 Hz, 1H), 7.28 (dd, *J* = 8.7, 2.3 Hz, 1H), 7.18 (dd, *J* = 2.4, 0.8 Hz, 1H), 7.07 (d, *J* = 8.4 Hz, 1H), 6.91 (dd, *J* = 8.8, 0.9 Hz, 1H), 3.97 (q, *J* = 7.0 Hz, 2H), 1.36 (t, *J* = 7.0 Hz, 3H).

#### 3.2.3. Synthesis of (*E*)-10-ethyl-7-(2-(pyridin-4-yl)vinyl)-10H-phenothiazine-3-carbaldehyde (EP)

Compound **2** (3.0 g, 9.0 mmol) and 4-vinylpyridine (1.4 g, 13.5 mmol) dissolved in toluene was refluxed with palladium(II) acetate (202.0 mg, 0.9 mmol), tri(o-tolyl)phosphine (410.0 mg, 1.4 mmol), and trimethylamine (5.0 mL, 35.8 mmol) for 72 h in N_2_ atmosphere. The crude mixture was concentrated under reduced pressure. Silica gel column chromatography was run to purify the product by using n-hexane and EA (*v*/*v* = 1:3). ^1^H NMR (400 MHz, acetone-d6) δ 9.82 (s, 1H), 8.52 (d, *J* = 5.6 Hz, 2H), 7.72 (dd, *J* = 8.5, 1.9 Hz, 1H), 7.58 (d, *J* = 1.9 Hz, 1H), 7.51–7.38 (m, 7H), 7.20–7.06 (m, 3H), 4.09 (q, *J* = 7.0 Hz, 2H), 1.43 (t, *J* = 7.0 Hz, 3H); ^13^C NMR (101 MHz, Acetone) δ 190.97, 151.59, 150.75, 145.99, 144.67, 133.61, 133.06, 132.75, 131.60, 128.86, 128.52, 126.58, 126.44, 124.75, 124.56, 122.04, 117.44, 116.47, 43.68, 13.50.

#### 3.2.4. Synthesis of 4-((1*E*,3*E*)-4-(benzo[d]thiazol-2-yl)buta-1,3-dien-1-yl)-*N*,*N*-dimethylaniline (Compound **3**)

2-Methylbenzothiazole (2.0 g, 13.7 mmol), 4-(Dimethylamino)cinnamaldehyde (2.0 g, 11.4 mmol) and potassium tert-butoxide (1.5 g, 13.7 mmol) were mixed in 30 mL DMF at 80 °C overnight. Fifty milliliters (50 mL) of deionized water was added to the crude mixture to obtain orange precipitate. The precipitate was collected by vacuum filtering and purified by recrystallization in ethanol. ^1^H NMR (400 MHz, chloroform-d) δ 7.98–7.95 (m, 1H), 7.87–7.82 (m, 1H), 7.46 (ddd, *J* = 8.3, 7.2, 1.3 Hz, 1H), 7.42 (d, *J* = 8.9 Hz, 2H), 7.38–7.34 (m, 2H), 6.89 (s, 1H), 6.86–6.83 (m, 2H), 6.71 (d, *J* = 8.9 Hz, 2H), 3.04 (s, 6H).

#### 3.2.5. Synthesis of 3-(4-boronobenzyl)-2-((1E,3E)-4-(4-(dimethylamino)phenyl)buta-1,3-dien-1-yl)benzo[d]thiazol-3-ium bromide (BT) was Synthesized by the Method Reported [12]

Compound **3** (1.0 g, 3.3 mmol) and 4-(bromomethyl) phenylboronic acid (0.6 g, 3.0 mmol) were mixed in MeCN/THF (*v*/*v* = 1:1) under nitrogen atmosphere. The reaction was heated to 80 °C for two days. The crude mixture was concentrated under reduced pressure and purified by recrystallization in DCM to obtain dark violet product. ^1^H NMR (400 MHz, DMSO-d6) δ 8.37 (dd, *J* = 7.9, 1.3 Hz, 1H), 8.12 – 8.04 (m, 4H), 7.79 (d, *J* = 8.0 Hz, 2H), 7.75–7.67 (m, 2H), 7.55 (d, *J* = 8.9 Hz, 2H), 7.50 (d, *J* = 14.8 Hz, 1H), 7.38 (d, *J* = 14.4 Hz, 1H), 7.25 (d, *J* = 8.1 Hz, 2H), 7.17 (dd, *J* = 14.9, 11.1 Hz, 1H), 6.79 (d, *J* = 9.0 Hz, 2H), 6.01 (s, 2H), 3.05 (s, 6H). ^13^C NMR (101 MHz, DMSO) δ 171.75, 152.88, 149.77, 141.74, 135.92, 135.26, 131.36, 129.75, 128.23, 127.88, 126.15, 124.74, 123.32, 116.58, 112.67, 111.82, 51.25. Q-TOF *m*/*z*: calcd, 397.1733 [M − Br − BO2H2]^+^; found, 397.1718.

### 3.3. Preparation of ONOO^−^ and Other Reactive Oxygen Species

ClO^−^, H_2_O_2_, NO·, NO_2_^−^, NO_3_^−^, and cysteine obtained from commercial sources were diluted or dissolved in water. ONOO^-^ was prepared by mixing pre-cooled 0.6 M NaNO_2_, 0.6 M HCl, and 0.7 M H_2_O_2_ into 3 M NaOH at 0 °C. Manganese dioxide was then added to the ONOO^−^ solution to eliminate the residual H_2_O_2_ and was removed by simple filtration. The ONOO^−^ solution was stored at −80 °C. The concentration of peroxynitrite was estimated by its extinction coefficient of 1670 M^−1^ cm^−1^ at 302 nm before use [19,20].

### 3.4. Optical Response of PB-PVA to ONOO^−^

The absorption and emission spectra of the probe at different concentrations of ONOO^−^ were measured at room temperature. The probe was measured 3 min after the addition of ONOO^−^. The absorption spectra were scanned from 300 to 800 nm while the emission spectra were scanned from 510 to 800 nm. The ratio between the emission intensities of two peaks from the probe when exposed to different concentrations of ONOO^−^ was calculated in order to investigate the relationship between the ratio and the concentration of ONOO^−^ exposed. The probe was also tested for its selectivity towards ONOO^−^ by investigating the fluorescence intensity ratios when exposed to various ROS. The time course of the probe response to ONOO^−^ was measured by tracking the fluorescence intensity at 600 nm with 10 s interval.

### 3.5. Cell Culture and Confocal Cell Imaging

HeLa cells were obtained from the Faculty of Health Sciences, University of Macau. The HeLa cells were cultured in RPMI-1640 medium supplemented with 10% of fetal bovine serum (FBS) and 1% of penicillin-streptomycin solution at 37 °C and 5% CO_2_ in a humidified environment. MTT assay was performed to evaluate the probe cytotoxicity with the method mentioned [21]. For confocal imaging of exogenous ONOO^−^, the cells were incubated with the probe (5 μM) for at least 2 h. DMSO (1% *v/v*) was added to culture medium to increase the solubility of probe. The cells were washed with PBS and further incubated with SIN-1 dissolved in fresh culture medium (1 mM) for 1 h. The treated HeLa cells were observed with a Carl Zeiss LSM710 confocal microscope.

## 4. Conclusions

In conclusion, we have developed a polymeric fluorescent probe PB-PVA for ratiometric measurement of ONOO^−^ and imaging of ONOO^−^ in living cells. This cell-permeable probe displays good ratiometric detection of ONOO^−^ at low concentration with good selectivity and fast response. These properties could allow the probe to be utilized in ONOO^−^ related in vitro studies as a promising measuring instrument in the future.

## Supplementary Materials

The following are available online. Figure S1. Probe fluorescence response to wide range of concentration of ONOO^−^ from 0 to 80 μM; Figure S2. The absorption spectrum of the probe before and after the addition of ONOO^−^ (10µM) in DMSO; Figure S3. ^1^H NMR of Compound 1; Figure S4. ^1^H NMR of Compound 2; Figure S5A. ^1^H NMR of EP; Figure S5B. ^13^C NMR of EP; Figure S5C. Q-TOF MS of EP; Figure S6. ^1^H NMR of P-PVA; Figure S7. ^1^H NMR of PB-PVA.
