# Supplementary material for: Ratiometric Polymer Probe for Detection of Peroxynitrite and the Application for Live-Cell Imaging
Source: Molecules. 2019 Sep 24;24(19):3465. doi: 10.3390/molecules24193465 (PMC6804088; doi:10.3390/molecules24193465)
Supplement: Supplementary file 1 [file molecules-24-03465-s001.pdf]

Supporting Information

Ratiometric Polymer Probe for Detection of Peroxynitrite and the Application for Live-Cell Imaging

Hio Kuan Lao, Jingyun Tan, Chunfei Wang and Xuanjun Zhang*

Cancer Centre and Centre of Reproduction, Development and Aging, Faculty of Health Sciences, University of Macau, Macau SAR, 999078, P. R. China; cb52929@connect.um.edu.mo (H.K.L.); yb57620@connect.um.edu.mo (J.T.); yb67596@connect.um.edu.mo (C.W.)

* Correspondence: yb57620@connect.um.edu.mo (J.T.); xuanjunzhang@um.edu.mo (X.Z.)
Tel.: +853-8822-4928

Table of Contents

1. Absorption spectral response of the probe PB-PVA 2
2. Characterization of intermediates and the probe PB-PVA 3

1. **Absorption Spectral Response of the Probe PB-PVA**

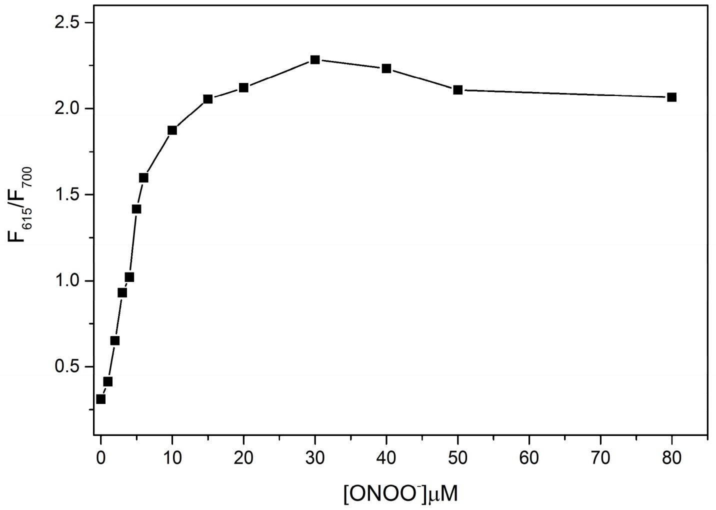

Figure S1. Probe fluorescence response to a wide range of concentration of $ONOO^-$ from 0 to 80 μM.

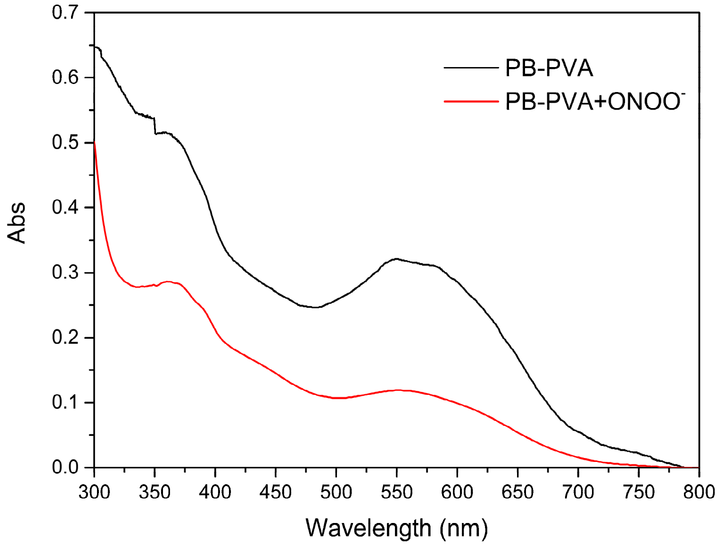

Figure S2. The absorption spectrum of the probe before and after the addition of $ONOO^-$(10 μM) in DMSO.

2. Characterization of intermediates and the probe PB-PVA

Compound 1 and 2 were characterized by ^{1}H NMR (Figures S3–S4).
EP was characterized by ^{1}H NMR, ^{13}C NMR, and MS (Figure S5A-C).
P-PVA was characterized by ^{1}H NMR (Figure S6).
PB-PVA was characterized by ^{1}H NMR (Figure S7).

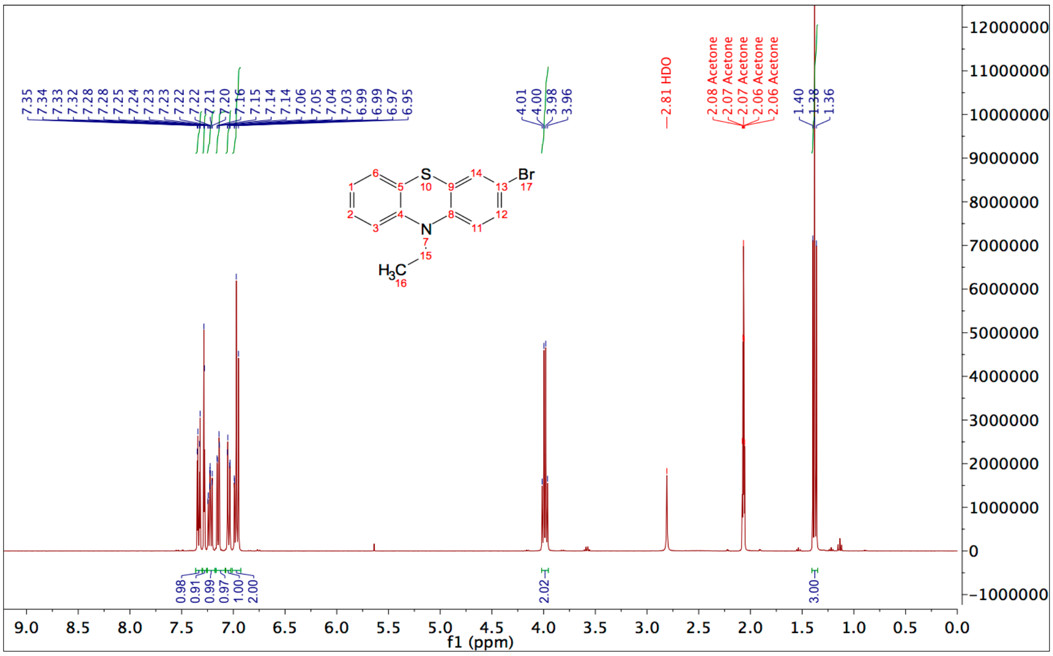

Figure S3. ^{1}H NMR of Compound 1.

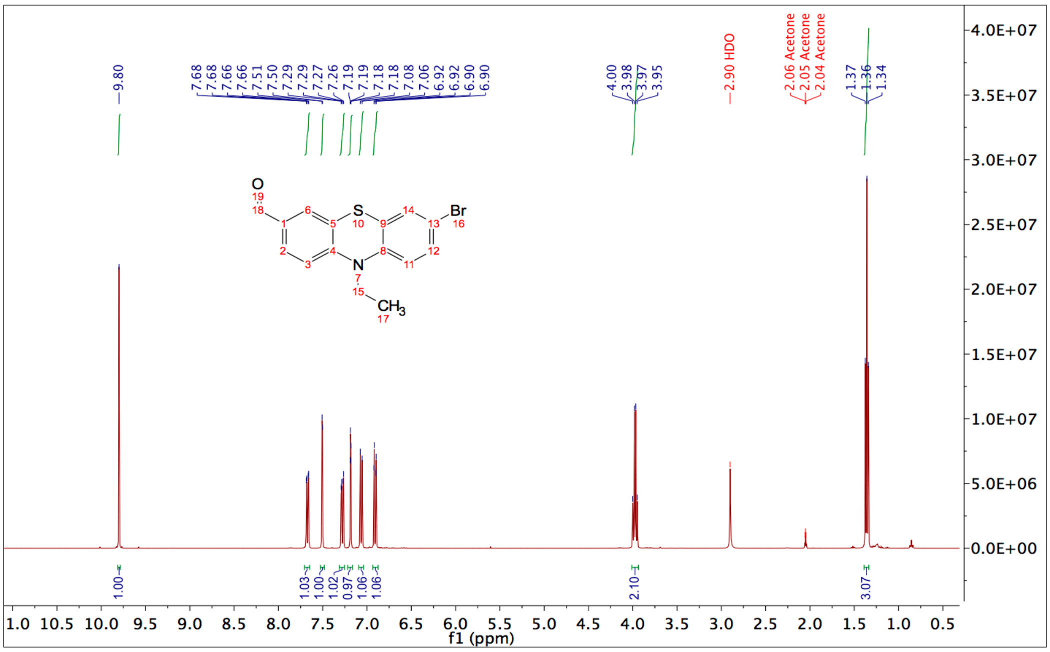

Figure S4. ^{1}H NMR of Compound 2.

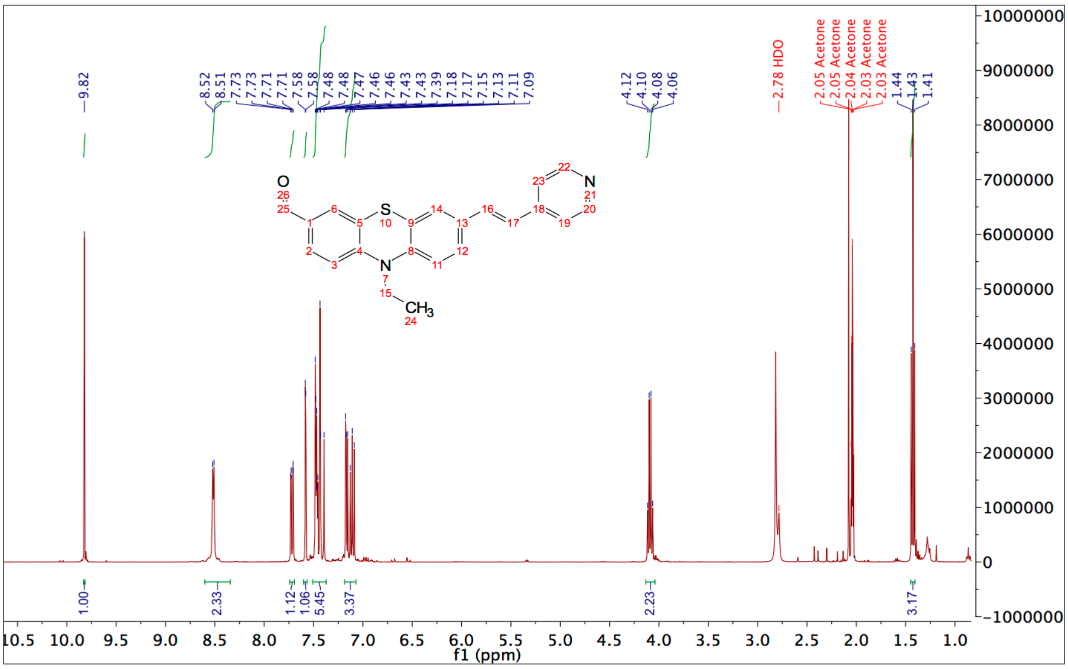

Figure S5A. ^{1}H NMR of EP.

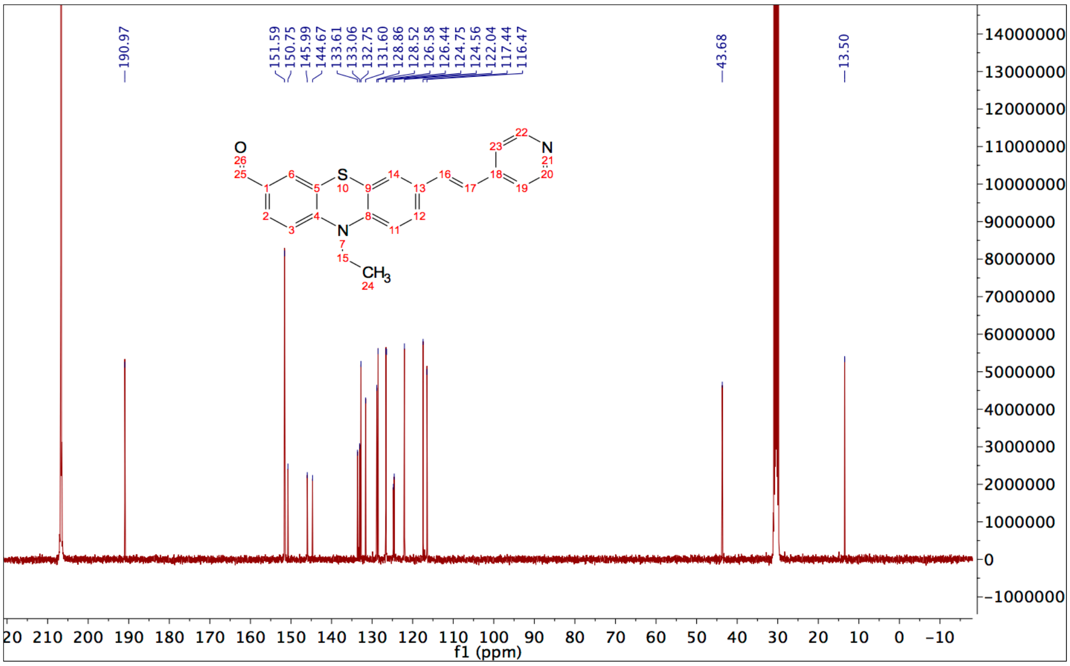

Figure S5B. ^{13}C NMR of EP.

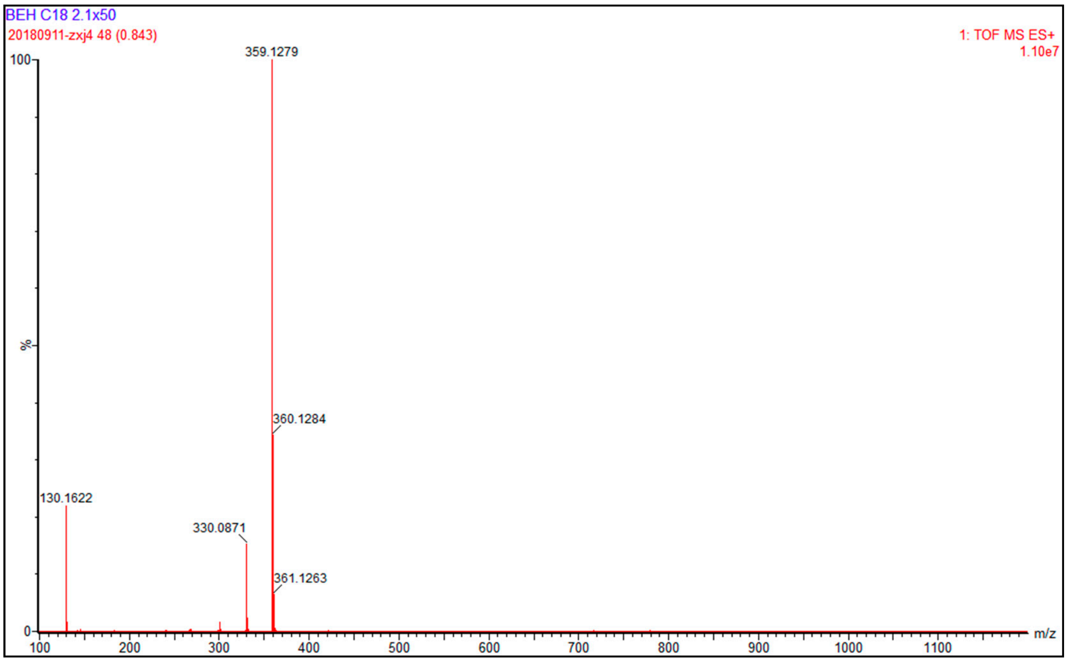

Figure S5C. Q-TOF MS of EP. The molecular weight of EP is 358.46.

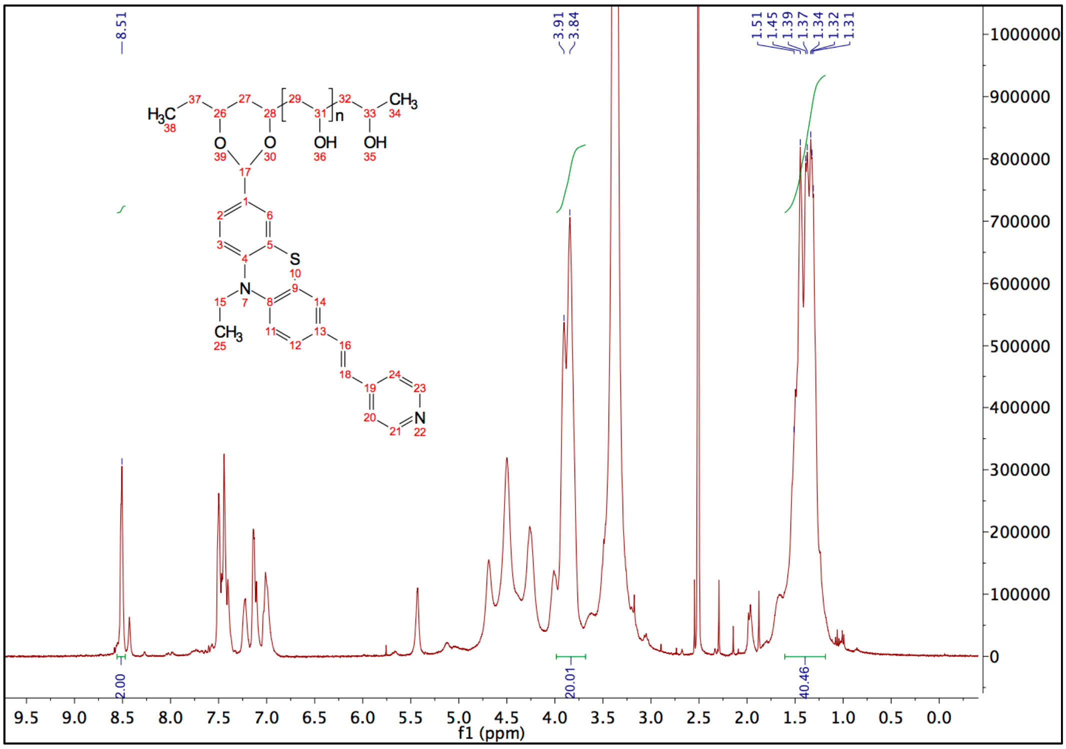

Figure S6. ^{1}H NMR of P-PVA. The ratio of EP grafted to the corresponding monomer is 1:10.

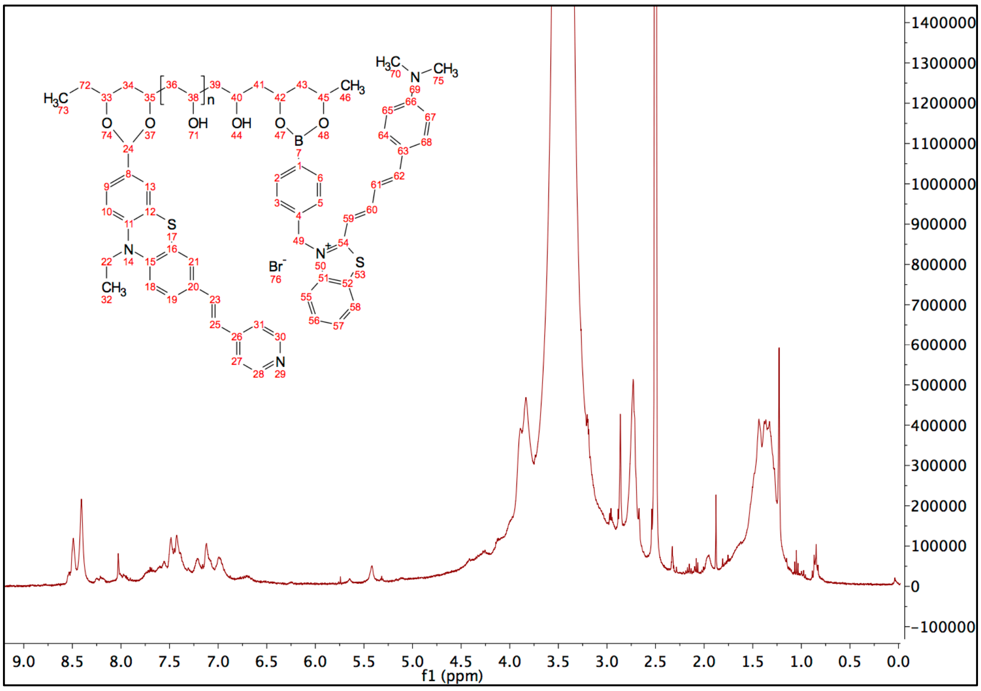

Figure S7. ^{1}H NMR of PB-PVA.
